# A study on mutation points of GDF9 gene and their association with prolificacy in Egyptian small ruminants

**DOI:** 10.1186/s43141-021-00181-8

**Published:** 2021-06-07

**Authors:** Dalia M. Aboelhassan, Ahmed M. Darwish, Neama I. Ali, Inas S. Ghaly, Ibrahim M. Farag

**Affiliations:** Cell Biology Department, Genetic Engineering and Biotechnology Research Division, National Research Centre, 33 El Bohouth st., Dokki, Giza, 12622 Egypt

**Keywords:** GDF9 gene, Gene markers, T-ARMS-PCR, Prolificacy, Egyptian sheep and goats

## Abstract

**Background:**

Genetic variants of the GDF9 gene were considered to be the potent gene markers for improving fecundity traits in Egyptian sheep and goats. Also, these favorable gene variants could be applied in the breeding program by gene-assisted selection (GAS), aiming towards the potential amelioration of reproduction and production in such small ruminants. The present investigation was designed to evaluate the genetic variants of the GDF9 gene on fecundity traits including the mean number of lambing “MNL” and mean number of twin production “MNTP” of Egyptian sheep and goats.

**Results:**

This experiment involved 113 mothers, 83 of sheep and 30 of goats, at first, second, third, and fourth parity, and also 26 young females, 12 of sheep and 14 of goats at age of sexual maturation. T-ARMS-PCR analysis was performed on five mutation points (G1, G4, G6, G7, and G8). In sheep, the heterozygous mothers of G4 had significant elevation (*P* ≤ 0.05) of MNL and MNTP than wild-type homozygous ewes. However, the heterozygous mothers of G1 and G6 gave a reduction of MNL and MNTP as compared to mothers with wild-type genotypes. The ewes of G7 had heterozygous genotype (AG), and the ewes of G8 had wild type (CC). In goat, G4 and G7 were polymorphic, and G1, G6, and G8 were monomorphic type. Based on these findings, it must be selected the young sheep females of heterozygous in G4, and the young goat females of heterozygous in G4 and G7 for participating in a successful breeding program, because they will have potential high fecundity traits.

**Conclusion:**

The present results confirmed that the genetic variants of the GDF9 gene were considered to be the major gene markers for enhancement of the prolificacy in Egyptian sheep and goats and could be applied in a successful breeding program by gene-assisted selection (GAS) in small ruminants.

## Background

Sheep and goat species are considered to be small and litter**-**bearing animals. These small ruminants are ideally suited as the model organisms of farm animals for studying genes that involve in mechanisms controlling reproductive processes especially ovulation rate [3, 26, 28]. It has been established that an animal has an increase of ovulation rate; it consequently produces multiple births of twin or triplet. This animal can contribute more than 1.5 to 2.0 times towards meat production than the animal producing single offspring per lambing or kidding [3, 29]. Therefore, the improvement of reproductive traits has been of increasing interest in such small ruminants. Genetic variations of some genes had been documented to be associated with ovulation rate in sheep and goats [1, 28]. Prolificacy genetics in sheep [10, 13] or goats [1] emphasize the importance of major fecundity gene, namely growth differentiation factor 9 (GDF9) or Fec G. GDF9 gene was found to be a member of transforming growth factor-ß (TGF-ß) superfamily. It was mapped on ovine chromosome 5, spanning approximately 2.5 Kb and comparised two exons (exon 1397 bp and exon 2965 bp) and one intron (1126 bp) [30]. This gene was revealed to have an important role during early folliculogenesis in female reproduction as a growth and differentiation factor secreted by oocytes in mammals [14]. The expression of the GDF9 gene in the oocyte was observed at the primary follicular stage and continues through ovulation [12]. In ovine [12] and caprine [33], the expression of GDF9 mRNA and protein had been detected at all stages of ovarian follicles and luteal tissue.

According to the important role of the GDF9 gene in the folliculogenesis, the availability of its polymorphism could be very useful in the study of animal reproduction genetics and physiology [32]. The genetic polymorphisms of the GDF9 gene were revealed to affect fecundity traits in the animals, where the heterozygous genotypes were found to cause increased ovulation rate and consequently led to enhancement of the prolificacy in the farm animals, in comparison to wild-type homozygous mothers [4, 16, 18, 22]. These findings had been established in sheep [7, 9, 18, 25, 28] and goats [11, 19], whereas the heterozygous carrier mothers exhibited high fecundity traits by producing lambs (or kids) more than the wild-type animals or non-carriers.

According to Hanrahan et al. [18] and Khodabakhshzadeh et al. [20], eight variants (G1 to G8) of the GDF9 gene had been discovered by PCR and sequences in Cambridge and Belclare sheep. In this study, three nucleotide changes of the eight polymorphisms did not alter amino acids (G2, C→T, 471; G3, G→A, 477 and G5, A→G, 978 coding bases). However, the remaining five mutations (G1, G4, G6, G7, and G8) resulted in amino acid changes. Four mutations were induced in G1 (G→A in 260 coding base), G4 (A→G in coding base 721), G6 (G→A in 994 coding base), and G7 (G→A in 1111 coding base) causing translation of Arg to His (R87H), Glu to Lys (E241 K), Val to Ile (V332 I), and Val to Met (V371 M), respectively. Moreover, G8 variant [C (wild type allele) →T (mutant allele) in 1184 coding base] was found to cause the translation of Serine to Phenylalanine (S395F). In addition, Polley et al. [28] clarified by using the tetra arm-PCR (T-ARMS-PCR) technique the mutation points of GDF9 gene in prolific Garole sheep and observed five ones including G1, G4, G6, G7, and G8 at five positions R87H, E241K, V332I, V371M, and S395F, respectively.

Polley et al. [27], Polley et al. [28] and Ahlawat et al. [1] reported that the induction of amino acid changes due to mutation points of the GDF9 gene might be the major determinant that influences prolificacy in the animals. So, the present study aimed to investigate the genetic polymorphisms of the GDF9 gene represented in five mutation points, G1, G4, G6, G7, and G8, and evaluate their effects on fecundity traits including the mean number of lambing “MNL” and the mean number of twin production “MNTP” in Egyptian sheep and goats.

## Methods

### Experimental animals and DNA isolation:

In this study, 139 females of five breeds of Egyptian sheep (Barki, Osseimi, Rahmani, Saudanez, and Awase) and five breeds of Egyptian goats (Zaraibi, Damascus, Boer, Saanen, and Barki) were used. These animals consisted of 113 mothers (83 of sheep and 30 of goats) and 26 young animals (12 of sheep and 14 of goats) at age of sexual maturity. For studying the association of identified genotypes of the GDF 9 gene with fecundity traits including MNL and MNTP, the animal mothers had been used. These mothers were at first, second, third, and fourth parity. Concerning the young females, these animals had been used for the same reproduction trait (MNL and MNTP) by investigating and selecting the animals that carry favorable gene markers (alleles) to contribute and participate in a successful breeding program. The flocks of sheep and goats were sourced from Animal Production Farms belonging to the Faculty of Agriculture (Cairo University), Nubaria, and Governmental Halayieb, Egypt. Around 6 to 10 ml of blood samples of each animal were collected. These samples were obtained aseptically from the jugular vein and immediately transported to the laboratory in an ice box. Then the isolation of genomic DNA was made from white blood cells using a standard procedure (phenol-chloroform extraction protocol) mentioned previously by Sambrook and Russell [31]. The concentration and quality of genomic DNA were assessed and stored at − 20 °C until use.

### Tetra-primer amplification refractory mutation system PCR (T-ARMS-PCR) for screening of polymorphism of fecundity GDF 9 gene

#### Primer designing

The used primers in this study were previously designed by Ye et al. [36], and they were based on ovine GDF9 (AF078545) gene. These primers were recorded in Table 1.

**Table 1:** The sequences of primers T-ARMS-PCR based amplification of GDF9 gene mutation points

The sequences of primers	Type of primer	Mutation points	Product size (bp)	
G1	IF(A)	334 CTGCAGCCAGATGACAGAGCTTTTCA 359	Mut	205
	IR(G)	388 CGTATGCCTTATAGAGCCTCTTCATGTCGC 359	Wt	247
	OF	168 GCCTGGCTCTGTTTTCCTATTAGCCTTG 188	Co	396
	OR	537 TCTTCTTCCCTCCACCCATTAACCAATC 510		
G4	IF(G)	296 TTCACATGTCTGTAAATTTTACATGTGAGG 325	Mut	212
	IR(A)	350 GCTGAAGGATGCTGCAGCTGGTCGTT 325	Wt	261
	OF	90 CAACAACTCCATTTCTTTTCCCTTTCCTG 118	Co	417
	OR	506 TAGGCAGATAGCCCTCTC TTCTGGTCAG 479		
G6	IF(A)	573 CAGCTCTGAATTGAAGAAGCCTCGGA 598	Mut	193
	IR(G)	625 ATTCACTCAGATTGACTGAAGCTGGCAC 598	Wt	223
	OF	403 TATCTGAACGACACAAGTGCTCAGGCTT 430	Co	362
	OR	764 CTGGGACAGTCCCCTTTACAGTATCGAG 737		
G7	IF(A)	688 AGTCAGCTGAAGTGGGACAACTGGAGTG 715	Mut	187
	IR(G)	742 ATCGAGGGTTGTATTTGTGTGGGGCAAT 715	Wt	212
	OF	556 AGAGACCAGGAGAGTGCCAGCTCTGAAT 583	Co	343
	OR	898 CGATGGCCAAAACACTCAAAGGGCTATA 871		
G8	IF(T)	763 AGGGCGGTCGGACATCGGTATGGATT 788	Mut	146
	IR(C)	817 TGATGTTCTGCACCATGGTGTGAACCGTAG788	Wt	108
	OF	710 GGATTGTGGCCCCACACAAATACAACCC 737	Co	198
	OR	907 CATCAGGCTCGATGGCCAAAACACTCAA 880		

*I* inner primers, *O* outer primers, *F* forward, *R* reverse, *Mut* size of mutant fragment, *WT* size of wild type fragment, *Co* size of control fragment

#### T-ARMS-PCR technique

This technique amplifies both wild-type and mutant alleles together with a control fragment in a single tube PCR reaction. Two allele-specific (inner) primers were designed in opposite orientation and in combination with the common (outer) primers that amplify both the wild-type and the mutant amplicons. In order to increase the specificity of the classical reaction of T-ARMS-PCR, a deliberate mismatch must be introduced at position-2 from the 3 termini of inner primers [21, 24]. Because the mutation point was found to be asymmetrically located with respect to the common (outer) primers, the allele-specific amplicons have different product length and could be easily separated by standard agarose gel electrophoresis.

#### PCR amplification and gel electrophoresis

The reaction of PCR has been performed in a 25-μl reaction volume containing 2.5 μl of thermophilic DNA polymerase 10x buffer (10 mM Tris - HCL (pH 9.0 at 25 °C), 50 mM KCL and 0.1% Triton X-100), 200 μM of dNTPs, 25 mM MgCl_2_, 10 pmol of each primer and 0.5 units of Taq DNA polymerase (Promega Corporation, Madison, WI, USA), and around 50 to 100 ng of genomic DNA that has been used as a template. The cycling conditions of PCR parameters for each allele had been optimized separately for detecting each of the allele-specific amplification. The amplifications of PCR for GDF9 gene were performed as follows with a simple program: the cycling conditions were 4 min at 94 °C, 32 cycles of 30–45 s at 94 °C, annealing temperature ranging from 50 to 56 depending upon mutation point, 30 s at 72 °C, and a final cycle for 4–6 min at 72 °C. The products of PCR had been separated by horizontal submarine agarose (2% free from DNase and RNase) electrophoresis in 1x TAE buffer at 80v. Then, the gel was stained with ethidium bromide solution (0.5 μg/ml) for 10 min in darkness and photographed using a molecular imager (Gel Doc XR, BIO-RAD).

### Statistical analysis

The data on fecundity traits had been statistically analyzed by one-way ANOVA followed by two-way ANOVA. Among mothers, the variations were significantly evaluated according to the method of Waller and Duncan [34]. The frequencies of alleles were compared using the same ANOVA analyses. All values were reported as mean ± S.E. and the differences were considered significant at a *P* value ≤ 0.05.

## Results

**Fig. 1:**
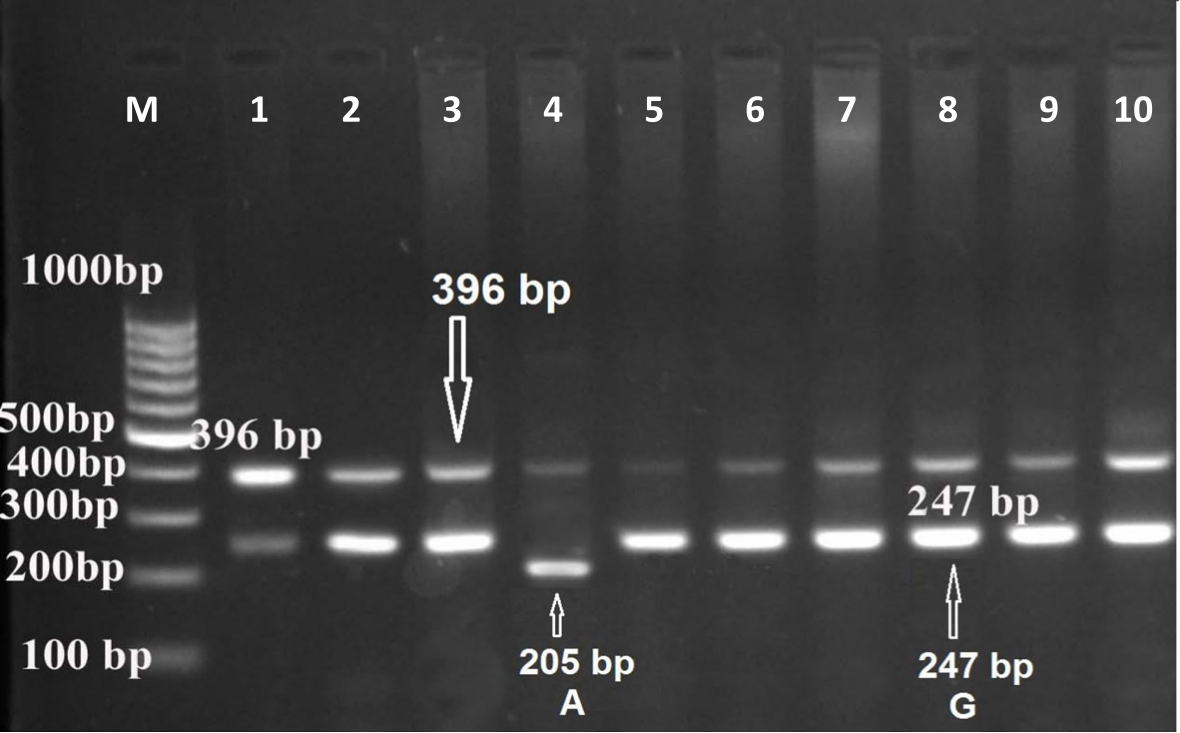
Electrophoresis of agarose gel (2%) of polymerase chain reaction (PCR) product of tetra-primer amplification refractory mutation system- PCR for G1 locus of GDF9 gene. The Lanes of 1 to 10: Amplification of wild type or heterozygous in a mother sheep genomic DNA. Sizes of common outer product (396 bp) and allele-specific wild type (247 bp) and mutant-type (205 bp) inner product are indicated by an arrow of the respective gel photograph. Lane M: DNA molecular weight marker (O` Range Ruler 100 bp DNA Ladder, Fermentas, Lithuania). G= wild type allele, A= mutant allele

**Fig. 2:**
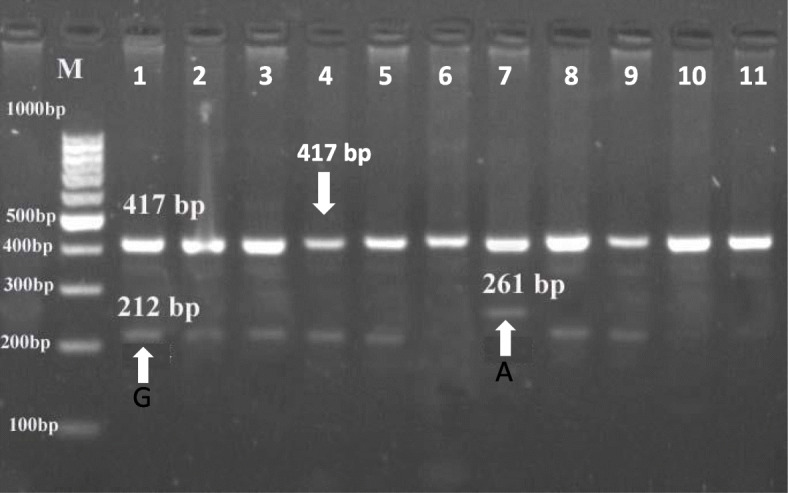
Electrophoresis of agarose gel (2%) of polymerase chain reaction (PCR) product of tetra-primer amplification refractory mutation system- PCR for G4 locus of GDF9 gene. The Lanes of 1 to 11: Amplification of wild type or heterozygous in a mother sheep genomic DNA. Sizes of the common outer product (417 bp) and allele-specific wild type (261 bp) and mutant-type (212 bp) inner product are indicated by an arrow of the respective gel photograph. Lane M: DNA molecular weight marker (Ơ Range Ruler 100 bp DNA Ladder, Fermentas, Lithuania). A= wild type allele, G= mutant allele

**Fig. 3:**
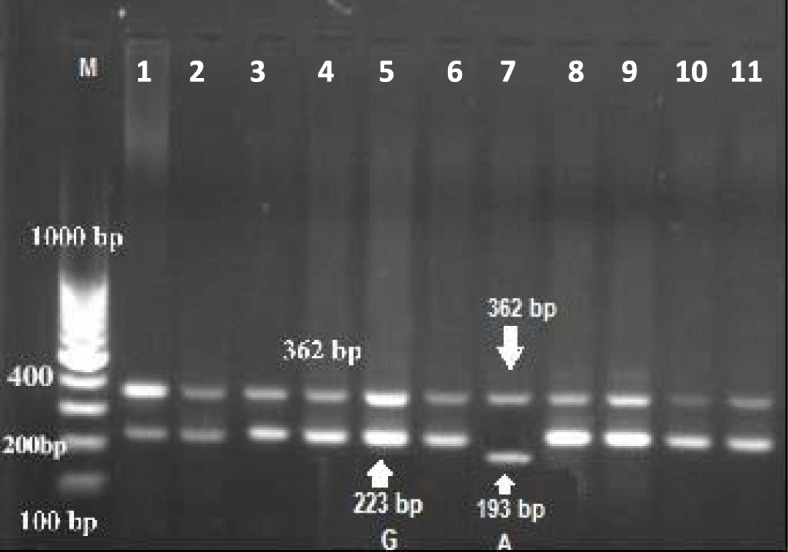
Electrophoresis of agarose gel (2%) of polymerase chain reaction (PCR) product of tetra-primer amplification refractory mutation system- PCR for G6 locus of GDF9 gene. The Lanes of 1 to 11: Amplification of wild type or heterozygous in a mother sheep genomic DNA. Sizes of the common outer product (362 bp) and allele-specific wild type (223 bp) and mutant-type (193 bp) inner product are indicated by an arrow of the respective gel photograph. Lane M: DNA molecular weight marker (O` Range Ruler 100 bp DNA Ladder, Fermentas, Lithuania). G = wild type allele, A = mutant allele

**Fig. 4:**
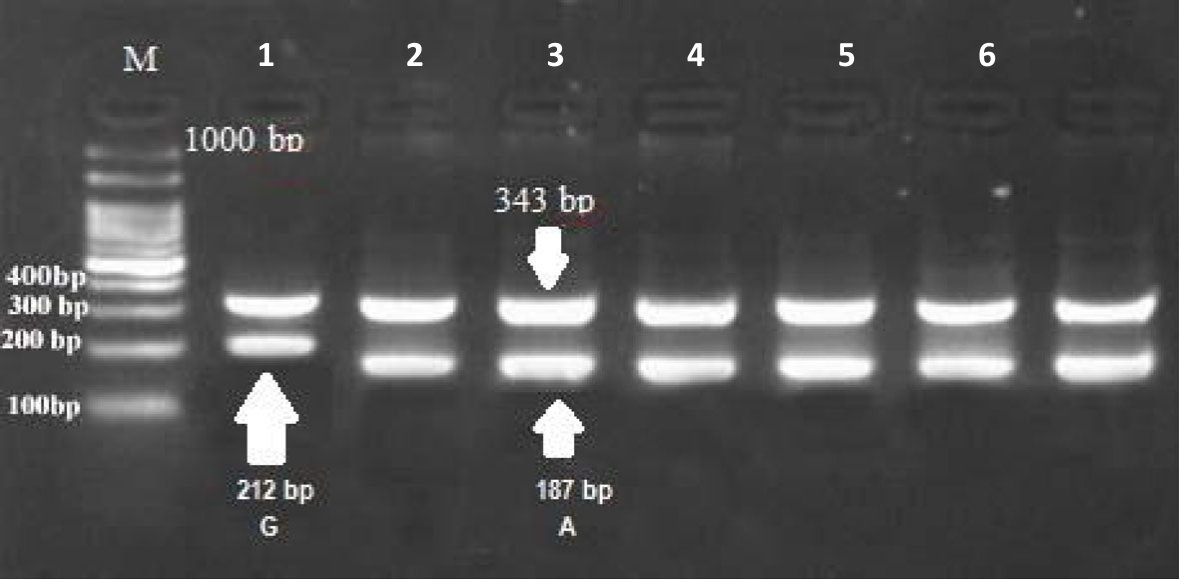
Electrophoresis of agarose gel (2%) of polymerase chain reaction (PCR) product of tetra primer amplification refractory mutation system- PCR for G7 locus of GDF9 gene. The Lanes of 1 to 7: Amplification of wild type or heterozygous in a mother goat genomic DNA. Sizes of the common outer product (343 bp) and allele-specific wild type (212 bp) and mutant-type (187 bp) inner product are indicated by an arrow of the respective gel photograph. Lane M: DNA molecular weight marker (O` Range Ruler 100 bp DNA Ladder, Fermentas, Lithuania). G = wild type allele, A = mutant allele

**Fig. 5:**
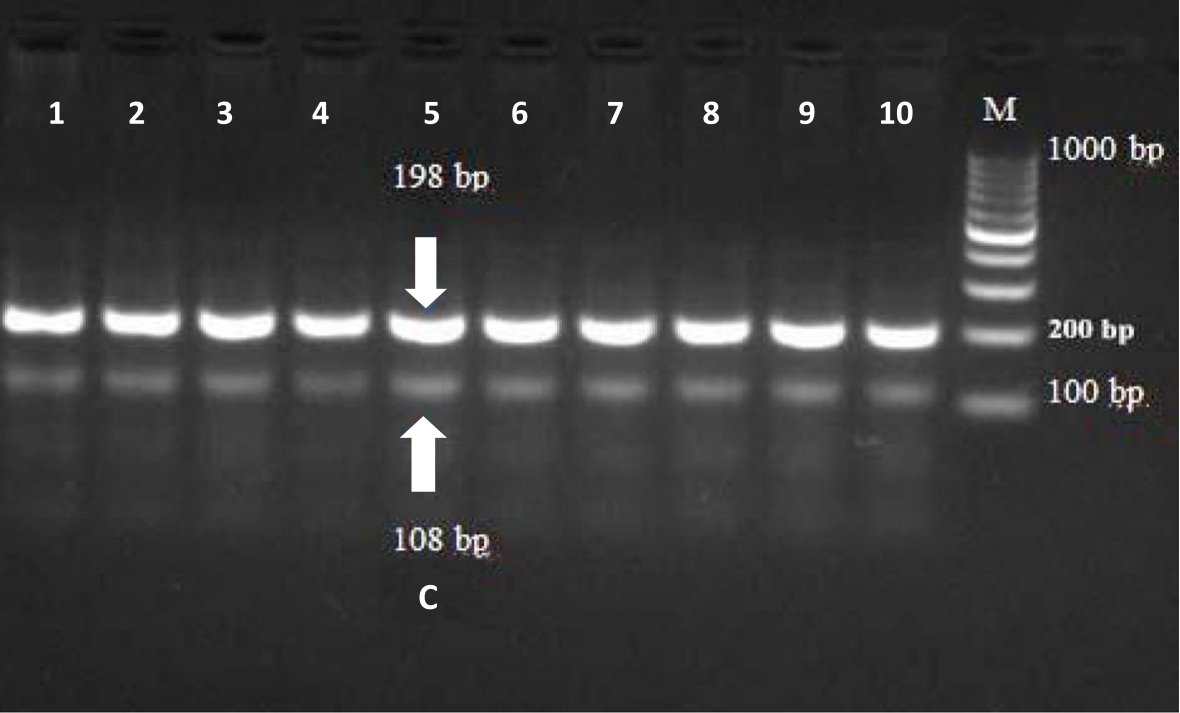
Electrophoresis of agarose gel (2%) of polymerase chain reaction (PCR) product of tetra-primer amplification refractory mutation system- PCR for G8 locus of GDF9 gene in sheep and goats. The Lanes of 1 to 10: Amplification of common outer product (198 bp) and allele-specific wild type (108 bp) are indicated by an arrow of the respective gel photograph. Lane M: DNA molecular weight marker (O` Range Ruler 100 bp DNA Ladder, Fermentas, Lithuania). C = wild type allele

In the present study, the method of tetra-primer ARMS-PCR had been successfully applied to type a total of five mutation points of the GDF9 gene. These mutation points viz G1, G4, G6, G7, and G8 were analyzed (Figs. 1, 2, 3, 4, and 5) in Egyptian sheep and goat breeds.

### T-ARMS-PCR analysis in sheep breeds:

The results revealed polymorphic type in G1, G4, and G6 mutation points, where wild-type (GG in G1 and G6 or AA in G4) and heterozygous (AG) in the above three mutation points had been observed (Table 2). The genotypes and allele frequencies had been identified in Table 3. The results on G1 and G6 were identical.

**Table 2:** The effect of mutation points of GDF9 gene on fecundity traits in Egyptian sheep

Species	Total No. of animals	No. of FSH	No. of mothers	Allele name or mutation point (M.P.)		No. of FSH with M.P	% of FSH with M.P	No. of mothers with M.P	% of mothers with M.P	Type of birth		Mean No. of lambing MNL	Mean No. of twin production MNTP
										% S	% T		
**Sheep**	**95**	**12**	**83**	**G1**	**W. Homozygous** **(GG)**	**11**	**0.91**	**81**	**0.98**	**1.01**	**0.58**	**2.17 ± 0.44**^**b**^	**1.59 ± 0.44**^**b**^
					**Heterozygous** **(AG)**	**1**	**0.08**	**2**	**0.02**	**0.02**	**0.01**	**0.048 ± 0.037**^**a**^	**0.036 ± 0.03**^**a**^
				**G4**	**W. Homozygous** **(AA)**	**10**	**0.83**	**11**	**0.13**	**0.14**	**0.06**	**0.3 ± 0.18**^**a**^	**0.2 ± 0.14**^**a**^
					**Heterozygous** **(AG)**	**2**	**0.17**	**72**	**0.87**	**0.89**	**0.53**	**1.95 ± 0.55**^**b**^	**1.42 ± 0.42**^**b**^
				**G6**	**W. Homozygous** **(GG)**	**12**	**1.0**	**81**	**0.98**	**1.01**	**0.58**	**2.17 ± 0.44**^**b**^	**1.59 ± 0.44**^**b**^
					**Heterozygous** **(AG)**	**0.0**	**0.0**	**2**	**0.02**	**0.02**	**0.01**	**0.048 ± 0.037**^**a**^	**0.036 ± 0.03**^**a**^
				**G7**	**W. Homozygous** **(GG)**	**0.0**	**0.0**	**0.0**	**0.0**	**0.0**	**0.0**	**0.0**^**a**^	**0.0**^**a**^
					**Heterozygous** **(AG)**	**12**	**1.0**	**83**	**1.0**	**1.04**	**0.59**	**2.21 ± 0.44**^**b**^	**1.62 ± 0.38**^**b**^
				**G8**	**W. Homozygous** **(CC)**	**12**	**1.0**	**83**	**1.0**	**1.04**	**0.59**	**2.21 ± 0.44**^**b**^	**1.62 ± 0.38**^**b**^
					**Heterozygous** **(CT)**	**0.00**	**0.00**	**0.00**	**0.00**	**0.00**	**0.00**	**0.00**^**a**^	**0.00**^**a**^

*FSH* females at age of sexual maturation, *M.P* mutation point, *W. Homozygous* wild-type homozygous, *S.* single birth, *T.* twin birth

Data expressed as mean ± SE values followed by different superscript letters are significantly different from one another within the same columns (*P* ≤ 0.05)

**Table 3:** Genotype and allele frequencies in mutation points of GDF9 gene of Egyptian sheep

Mutation point	No. of animals (mothers + FSH)	Genotype frequencies		Allele frequencies	
G1	95	GG 0.97	AG 0.03	G 0.985 ± 0.11^**b**^	A 0.015 ± 0.0^**a**^
**G4**	**95**	**AA** **0.22**	**AG** **0.78**	**G** **0.39 ± 0.1**^**a**^	**A** **0.61 ± 0.08**^**b**^
**G6**	**95**	**GG** **0.98**	**AG** **0.02**	**G** **0.99 ± 0.07**^**b**^	**A** **0.01 ± 0.0**^**a**^
**G7**	**95**	**GG** **0.0**	**AG** **1.0**	**G** **0.5 ± 0.01**^**a**^	**A** **0.5 ± 0.08**^**b**^
**G8**	**95**	**CC** **1.0**	**CT** **0.0**	**C** **1.0 ± 0.05**^**b**^	**T** **0.0 ± 0.0**^**a**^

*FSH* females at age of sexual maturation

Data expressed as mean ± SE values followed by different superscript letters are significantly different from one another within the same columns (*P* ≤ 0.05)

Concerning the results on G4, in this mutation point, the mothers with heterozygous genotype (AG) discriminated with an increase in each of the mean number of lambing and mean number of twin production as compared to individuals with wild-type (AA) homozygous genotype (1.95 ± 0.55 and 1.42 ± 0.42 vs. 0.3 ± 0.18 and 0.2 ± 0.14, respectively). Statistical analysis proved significant variations (*P* ≤ 0.05) between the two genotypes for prolificacy or fecundity traits (mean number of lambing MNL and mean number of twin production MNTP). However, the heterozygous (AG) mothers of G1 and G6 mutation points gave a reduction of “MNL” and “MNTP” as compared to mothers with wild type (GG). This reduction was found to be due to the very little number of mothers (*n* = 2) with heterozygous (AG) genotype in relation to a high number of mothers (*n* = 81) with wild-type genotype.

The results of T-ARMS-PCR analysis on G7 and G8 mutation points observed that all sheep breeds had monomorphic type, where heterozygous (AG) genotype was only found in G7, whereas wild-type homozygous (CC) genotype was only detected in G8.

### T-ARMS-PCR analysis in goat breeds

The results of T**-**ARMS-PCR analysis (Table 4) showed in mutation points G1, G6, and G8, that all goat breeds were monomorphic type, where wild-type homozygous genotype (GG) was only observed, whereas in mutation points, G4 and G7, the polymorphic types have been detected. The genotype and allele frequencies had been identified in Table 5. In G4, 12 mothers of goat breeds had wild-type homozygous genotype (AA), whereas the remaining 18 mothers had heterozygous genotype (AG). The animals with heterozygous genotype had more mean number of lambing (or kidding) and more mean number of twin production than the animals with wild-type homozygous genotype (1.86 ± 0.27 and 1.26 ± 0.317 vs. 1.43 ± 0.44 and 0.86 ± 0.27, respectively), in spite of the statistical analysis that showed no significant differences between the two genotypes for the two fecundity traits (MNL and MNTP). Also, in G7, 26 mothers had heterozygous genotype (AG), whereas the remaining 4 mothers had wild-type homozygous (GG) genotype. The mothers with heterozygous genotype discriminated with the high prolificacy than wild-type homozygous animals, where the mean number of lambing MNL and the mean number of twin production MNTP were raised in heterozygous genotype than those in wild type homozygous genotype (3.03 ± 0.66 and 1.93 ± 0.4 vs. 0.26 ± 0.17 and 0.2 ± 0.1, respectively), and statistical analysis showed highly significant (*P* ≤ 0.01) differences between the two genotypes for the two fecundity traits.

**Table 4:** The effect of mutation points of GDF9 gene on fecundity traits in Egyptian goats

Species	Total No. of animals	No. of FSH	No. of mothers	Allele name or mutation point (M.P.)		No. of FSH with M.P	% of FSH with M.P	No. of mothers with M.P	% of mothers with M.P	Type of birth		Mean No. of lambing MNL	Mean No. of twin production MNTP
										% S	% T		
Goats	44	14	30	G1	W. Homozygous (GG)	14	1.0	30	1.0	0.97	1.17	3.3 ± 0.69^e^	2.13 ± 0.29 ^d^
					Heterozygous (AG)	0.0	0.0	0.0	0.0	0.0	0.0	0.0^a^	0.0^a^
				G4	W. Homozygous (AA)	2	0.14	12	0.4	0.33	0.57	1.43 ± 0.44^bc^	0.86 ± 0.27 ^b^
					Heterozygous (AG)	12	0.86	18	0.6	0.67	0.6	1.86 ± 0.27^cd^	1.26 ± 0.317^bc^
				G6	W. Homozygous (GG)	14	1.0	30	1.0	0.97	1.17	3.3 ± 0.69^e^	2.13 ± 0.29 ^d^
					Heterozygous (AG)	0.0	0.0	0.0	0.0	0.0	0.0	0.0^a^	0.0^a^
				G7	W. Homozygous (GG)	0.0	0.0	4	0.13	0.13	0.07	0.26 ± 0.17^ab^	0.2 ± 0.1^a^
					Heterozygous (AG)	14	1.0	26	0.87	0.83	1.1	3.03 ± 0.66^de^	1.93 ± 0.4^cd^
				G8	W. Homozygous (CC)	14	1.0	30	1.0	0.97	1.17	3.3 ± 0.69^e^	2.13 ± 0.29 ^d^
					Heterozygous (CT)	0.0	0.0	0.0	0.0	0.0	0.0	0.0^a^	0.0^a^

*FSH* females at age of sexual maturation, *M.P* mutation point, *W. Homozygous* wild-type Homozygous, *S.* single birth, *T.* twin birth

Data expressed as mean ± SE values followed by different superscript letters are significantly different from one another within the same columns (*P* ≤ 0.05)

**Table 5:** Genotype and allele frequencies in mutation points of GDF9 gene of Egyptian goats

Mutation point	No. of animals (mothers + FSH)	Genotype frequencies		Allele frequencies	
**G1**	**44**	**GG** **1.0**	**AG** **0.0**	**G** **1.0 ± 0.1**^**b**^	**A** **0.0 ± 0.0**^**a**^
**G4**	**44**	**AA** **0.32**	**AG** **0.68**	**G** **0.34 ± 0.1**^**a**^	**A** **0.66 ± 0.09**^**c**^
**G6**	**44**	**GG** **1.0**	**AG** **0.0**	**G** **1.0 ± 0.07**^**b**^	**A** **0.0 ± 0.0**^**a**^
**G7**	**44**	**GG** **0.09**	**AG** **0.91**	**G** **0.54 ± 0.0.06**^**a**^	**A** **0.46 ± 0.06**^**b**^
**G8**	**44**	**CC** **1.0**	**CT** **0.0**	**C** **1.0 ± 0.11**^**b**^	**T** **0.0 ± 0.0**^**a**^

*FSH* females at age of sexual maturation.

Data expressed as mean ± SE values followed by different superscript letters are significantly different from one another within the same columns (*P* ≤ 0.05)

## Discussion

In the present work, the technique of the tetra-primer ARMS-PCR (T-ARMS-PCR) was successfully applied to type a total of five known mutation points (G1, G4, G6, G7, and G8) of the GDF9 gene that had been associated with fecundity traits in sheep and goats. Also, in previous studies on Indian prolific black Bengal goats [27] and prolife Garole sheep [28], the T-ARMS-PCR method was successfully utilized for a description of the same five mutation points (G1, G4, G6, G7, and G8) of GDF9 gene. This technique is considered to be an efficient procedure for genotyping single nucleotide polymorphisms (SNPs) [36]. It can achieve and able to produce many gene markers (SNPs) in a single PCR without prior sequence knowledge of the genomes of interest, where the mutant and wild-type alleles as well as the control fragment can be amplified in a single tube PCR reaction [24, 36]. This because the designing of primers in this technique has been made to amplify fragments of different sizes for each allele band. Therefore, the size of PCR product with different length can easily be separated by simple agarose gel electrophoresis [1, 24, 28, 36]. So, T-ARMS-PCR is an economical method for SNP scoring and a very useful tool for large-scale SNP analysis [27, 36]. In the present study, T-ARMS-PCR analysis clarified genetic polymorphisms of the GDF9 gene in Egyptian sheep and goats, where polymorphic types including heterozygous and wild-type homozygous genotypes were together observed in sheep at G1, G4, and G6 mutation points and goat at G4 and G7 mutation points in mothers. Also, the genetic polymorphisms were detected in young females at age of sexual maturation (FSH) at G1 and G4 of sheep breeds and at G4 of goat breeds. Similarly, in several studies on sheep and goats, the GDF9 gene was found to display abundant genetic polymorphisms: Concerning sheep, in Cambridge and Belclare sheep breeds, Hanrahan, et al. [18] and Khodabakhshzadeh et al. [20] detected by PCR-SSCP, eight variants (G1 to G8) of GDF9 gene. Also, in four sheep breeds including Small Tail Han. White Suffolk, Texel, and Tibetan sheep, Chang et al. [5] determined by PCR-SSCP, G2 mutation of GDF9 gene. Moreover, in five sheep breeds including Tail Han, Poll Dorest, Suffolk, German Mutton Merino, and Chinese Merino, Chen et al. [6] identified by PCR-SSCP, G4 mutation of GDF9 gene. In Iran, Barzegari et al. [2] revealed G1 mutation of GDF9 gene in Moghani and Ghezel sheep breeds. In India, Polley et al. [27] also observed G1 mutation of the GDF9 gene in the Garole sheep breed. Polley et al. [28] clarified five mutation points (G1, G4, G6, G7, and G8) of the GDF9 gene by using the T-ARMS-PCR technique in prolific Garole sheep. Chu et al. [7, 9] displayed G3 mutation of GDF9 gene in Small Tail Han and Dorset sheep. Concerning goats, Wu et al. [35] and Feng et al. [15] reported three mutations, c. 423G ≥ A, c.959A ≥ C, and c. 1189C ≥ A of the GDF9 gene in Jining Grey, Liaoning Cashmere, and Boer goats. Zhang et al. [37] detected one mutation (c.959A > C) in Yangtse River Delta White and Huanghuai goats. Moreover, Chu et al. [7, 9] identified two mutations (c.183A > C and c.336C > T) with three genotypes AA, AB, and BB in five goat breeds including high prolificacy breed (Jining Grey “JG”) and low prolificacy breeds (Boer “BO,” Wendeng Dairy “WD,” Liaoning cashmere Lc and Beijing native goats “BN”).

### The effect of variants of GDF9 gene on reproduction (or fecundity traits) in sheep and goats

The present results revealed that the heterozygous genotype (AG) at G4 mutation point of sheep mothers and G4 and G7 mutation points of goat mothers was discriminated with high rates of fecundity traits as compared to wild type homozygous genotype (AA in G4 or GG in G7), where the best findings of MNL and MNTP were identified of heterozygous mothers. Similarly in a previous study, Hanrahan et al. [18] observed that heterozygous of G8 mutation point of GDF9 gene was associated with increased prolificacy in Belclare and Cambridge sheep. Their results found that heterozygous carrier mothers exhibited one to two additional ovulations when compared to non-carrier, where in Belclare ewes, the ovulation rates of heterozygous mutation and wild type were 2.67 ± 0.89 and 1.92 ± 0.28, respectively. Also, in Cambridge ewes, the ovulation rates of heterozygous mutation and wild type were 4.28 ± 0.31 and 2.27 ± 0.49, respectively.

In Guizhou white goats, Du et al. [11] detected heterozygous genotype (g.1189G > A mutation) of GDF9 gene in eight of 33 high prolificacy mothers, where these eight mothers were found to give 3 kids per litter rather than in 112 low prolificacy goats with wild type genotype. Also, in Guizhou black goats, Huang et al. [19] revealed a heterozygous genotype (c.1133C < T mutation) of the GDF9 gene that could increase the prolificacy in goats. Their results pointed that the two heterozygous mothers were found to give 3 kids per litter as compared to 12 of low prolificacy goats with wild-type genotype. In Thoka sheep, Nicol et al. [25] pointed in Fec TT mutation of GDF9 gene that mothers with heterozygous genotype produced 0.68 more lambs per ewe lambing than wild type animals. Moreover, in Small Tail Han Sheep, Chu et al. [7, 9] determined two genotypes, heterozygous (CD) and wild type (CC) of the GDF9 gene, and discovered that the mothers with heterozygous genotype (CD) had 0.77 (*P* < 0.05) lambs more than the mothers with wild-type homozygote genotype (CC). Our findings were also similar with that of Chu et al. [7, 9] who detected in Jining Grey goat does, genetic polymorphisms of the GDF9 gene and revealed that the mothers with heterozygous genotype (AB) had 0.56 (*P* < 0.01) kids more than those with wild type homozygous genotype (BB). Also, Feng et al. [15] observed in Jining Grey goats that heterozygous genotype (AC) of the GDF9 gene had been associated with high litter size in relation to wild-type genotype (AA), where their results proved that the mothers with heterozygous genotype (AC) had 0.63 (*P* < 0.01) kids more than mothers with wild-type homozygous genotype (AA). In a previous study on Moghani and Ghezel sheep, Barzegari et al. [2] found the obvious major effect of G1 mutation heterozygous of GDF9 gene on fecundity traits as compared to wild type or homozygous genotypes. Their findings clarified that seven ewes out of thirteen (53.8%) with G1 heterozygous gave twin birth, while five of 79 ewes (6.3) with wild-type gave twin birth as well as four ewes with homozygous genotype were observed to be fertile and all gave single birth.

The present results of T**-**ARMS-PCR analysis observed that the sheep mothers with heterozygous genotype (AG) of G1 and G6 mutation points had given a reduction of MNL and MNTP as compared to mothers with wild-type homozygous genotype (GG). This reduction was found to be due to the very little number of mothers (*n* = 2) with heterozygous genotype (AG) as compared to a high number of mothers (*n* = 81) with wild-type homozygous genotype (GG). However, in approximate similarity, Silva et al. [32] found a genetic variant of GDF9 in Brazilian Santa Ines sheep gene and detected that the homozygous ewes (EE) were fertile and had increases of ovulation rate (82%), twin pregnancy (44%) and prolificacy (58%) as compared to heterozygous mothers (+ E). Also in a previous study, Chu et al. [8] detected G3 mutation point of the GDF9 gene in Small Tail Han sheep and found that the litter size in the first and second parity of the mothers with homozygous genotype (AA) were 0.30 (*P* < 0.05) and 0.77 (*P* < 0.0001), respectively more than those of mothers with heterozygous (AB) genotype.

Furthermore, in Small Tail Han sheep, Gao [17] revealed genetic polymorphism of GDF9 gene and clarified that ewes with homozygous genotype (CC) had 0.63 (*P*< 0.05) lambs more than mothers with heterozygous genotype (CD). Melo et al. [23] reported in Brazilian Santa Ines sheep, that the mean average number of corpus luteum in the ewes with homozygous genotype (Fec G SI of mutation of GDF9 gene) was more than those of females with heterozygous genotype (2.4 ± 0.2 vs. 1.3 ± 0.1) after estrus synchronization.

Based on the present findings and above discussion, it must be selected the young sheep females (FSH) of heterozygous genotype in G4 mutation point as well as young goat females (FSH) of heterozygous genotype in G4 and G7 mutation points for participating in a successful breeding program, because these young animals will have potential high fecundity traits.

## Conclusion

In conclusion, according to the findings of genetic variants of GDF9 that showed G1, G4, and G7 mutation sites were polymorphic in sheep, in contrast to G8 which was monomorphic. While in goats, only mutation sites G4 and G7 were polymorphic whereas, G1, G6, and G8 were monomorphic. So, the present results confirmed that the genetic variants of the GDF9 gene were considered to be the major gene markers for enhancement of the prolificacy in Egyptian sheep and goats. Also, these favorable gene variants could be applied in a successful breeding program by gene-assisted selection (GAS), aiming towards the potential amelioration of reproduction and production in such small ruminants.

## Data Availability

The data and materials described in the manuscript, including all relevant raw data, will be freely available to any scientist wishing to use them for non-commercial purposes, without breaching.

## Abbreviations

GDF9: Growth differentiation factor 9
T-ARMS-PCR: Tetra-primer amplification refractory mutation system
